# Experimental and Numerical Investigation of 3D Printing PLA Origami Tubes under Quasi-Static Uniaxial Compression

**DOI:** 10.3390/polym14194135

**Published:** 2022-10-02

**Authors:** Weidong Chen, Chengjie Guo, Xiubin Zuo, Jian Zhao, Yang Peng, Yixiao Wang

**Affiliations:** 1 School of Textile and Material Engineering, Dalian Polytechnic University, Dalian 116034, China; 2 State Key Laboratory of Structural Analysis of Industrial Equipment, Dalian University of Technology, Dalian 116023, China

**Keywords:** polylactic acid, 3D printing, energy absorption, finite element simulation, brittle fracture

## Abstract

The investigation aims to study the effects of temperature and damage constitutive model on the energy absorption performance of polymeric origami tubes under quasi-static impact. The uniaxial tensile responses of 3D-printed polylactic acid (PLA) samples following standard ASTM-D412 have been studied to characterize the mechanical properties at three temperatures: 30 °C, 40 °C, and 50 °C. The damage constitutive model is used to accurately characterize the stress-strain relations of the PLA. Quasi-static compressive experiments are performed on polymetric tubes with different temperatures. The 3D-printed technique is used to ensure the integrated formation of these polymeric origami tubes. The user-defined material subroutine VUMAT for ABAQUS/Explicit has been developed for the damage model. Compared with the results, the observed deformation processes are well captured by the numerical simulations, and the influence of temperature on the axial compression is also analyzed in detail.

## 1. Introduction

**Figure 1:**
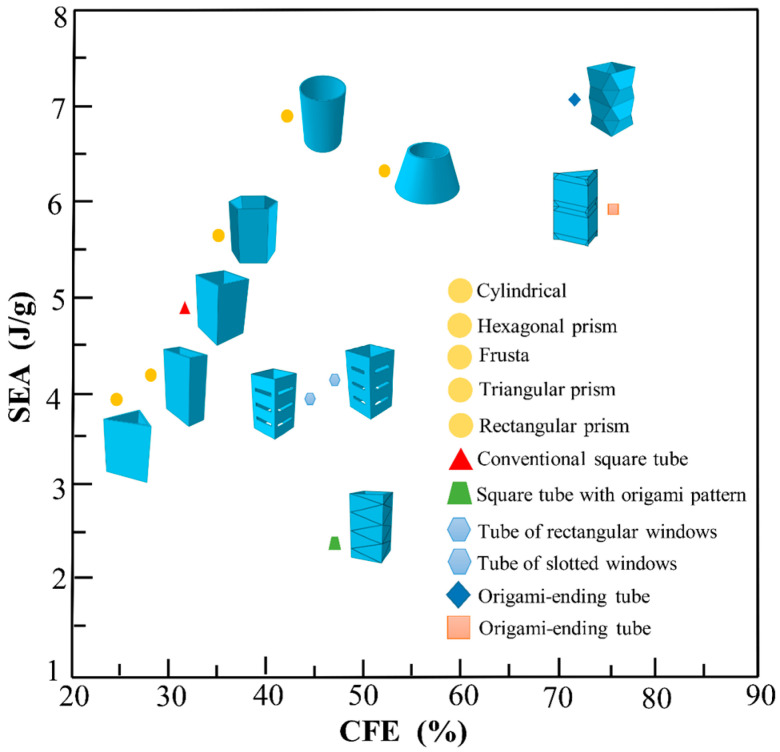
Comparison of *SEA* and *CFE* values of different structures.

In transport vehicle design, many efforts have been made in the past decades to improve the crashworthiness of the structure which is the ability to absorb impact energy in a controlled manner and decrease the injuries through crash events [1,2]. A common design approach to enhance the crashworthiness of a vehicle is to install energy absorption devices which deform and absorb kinetic energy during a low-speed collision. Due to light weight, excellent energy absorption, and low manufacturing costs, thin-walled tubes are widely used in vehicle engineering. Over the past few decades, a number of thin-walled tubes with different structures have been widely investigated, including corrugated tubes [3], folded tubes [4], grooved tubes [5], concave tubes [6], honeycomb structures [7], and lattice structures [8]. As one of the new types of thin-walled tubes [9], origami-based tubes have attracted considerable attention because they can be designed to guide the collapse following the pre-manufactured pattern and improve the energy absorption performance. Figure 1 compares the specific energy absorption (*SEA*) and crushing force efficiency (*CFE*) of various energy-absorbing boxes [10,11,12,13,14,15] with different structures. It can be seen from the figure that the *SEA* performance of origami crash box (OCB) is the best. In this paper, OCB is used to carry out research.

Extensive studies on thin-walled structures have so far been carried out through experimental, theoretical, and numerical methods. Wierzbicki and Abramowicz [16] presented a self-consistent theory and constructed a basic folding mechanism to obtain the mean crushing force of thin-walled rectangular columns. Modern materials such as plastic [17], metal [18], and fiber-reinforced composite [19,20] have been widely used to manufacture thin-walled tubes. Hanefi and Wierzbicki [21] derived the mean crushing force and the length of the local folding wave based on Alexander’s theory to investigate the collapse of externally composite reinforced metal tubes. Based on the super folding element theory, Chen and Wierzbicki [22] studied and derived the analytical model of multi-cell columns. The initial peak force and energy absorption have been heavily employed to evaluate the performance of thin-walled structures. Many researchers introduced different geometrical defects on the surface of the thin-walled tubes to minimize the initial peak and increase the energy absorption. Inspired by origami art, research group You [23,24] and Wang [25,26] developed a series of origami-based tubes to control the collapsed mode and meet crashworthiness criteria needs during crushing. Among origami-inspired structures introduced by research group Wang [27] was an OCB typical origami patterns, which will be further investigated in this paper.

It is noteworthy that the introduction of origami patterns to thin-walled tubes can increase manufacturing complexity. The traditional techniques to fabricate these structures consist of stamping and welding, which may cause structure asymmetry [28]. Therefore, the manufacturing processes play an important role in the mechanical performances of these structures. To ensure the integrated manufacture, Wang et al. [17] introduced the blow molding approach to fabricate the polymetric origami-based tubes to investigate the viscoelasticity of these structures. Meanwhile, the three-dimensional (3D) printing technique offers another route to manufacture origami tubes, which is still a topic of ongoing research. One of the most popular polymers used in 3D printing is PLA, which is undoubtedly regarded as a valuable bio-sourced polymer alternative in automotive industries [29,30]. The mechanical properties of 3D-printed parts made of PLA are strongly affected by the printed material, and the process parameters, such as layer thickness, printing speed, building temperature, fill style, support structure, printing direction, etc. To provide a basis for safety-critical design, great efforts have been made to investigate mechanical properties, in particular intra- and inter-layer strengths [31], tensile failure strength [32], and low-velocity impact response of PLA structures [33,34].

In this study, fused filament fabrication (FFF) 3D printing is used to fabricate the OCB by integrated formation. Experiments and numerical simulations are conducted to study the influence of temperature on the energy absorption of polymeric origami tubes under quasi-static uniaxial compression. The experiments showed that different constitutive models are needed to capture the complex true stress-strain behavior of 3D printing polylactic acid (PLA) material at different temperatures. A user-defined material subroutine VUMAT of ABAQUS is coded with the modified Mazars damage model. Numerical methods are constructed and validated with experimental results. The layout of the paper is as follows. Section 2 gives the materials and methods of origami tubes. Subsequently, the results and detailed discussions are presented in Section 3. Finally, conclusions are drawn in Section 4.

## 2. Materials and Methods

### 2.1. Geometry

**Figure 2:**
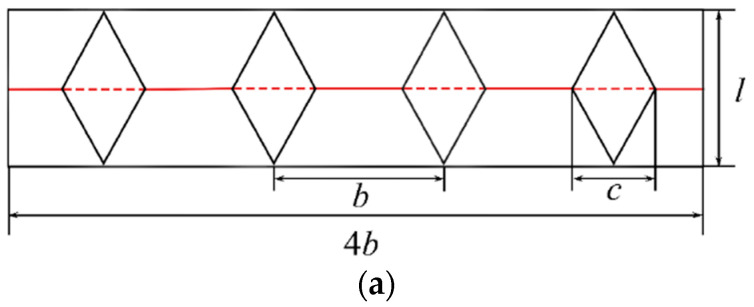

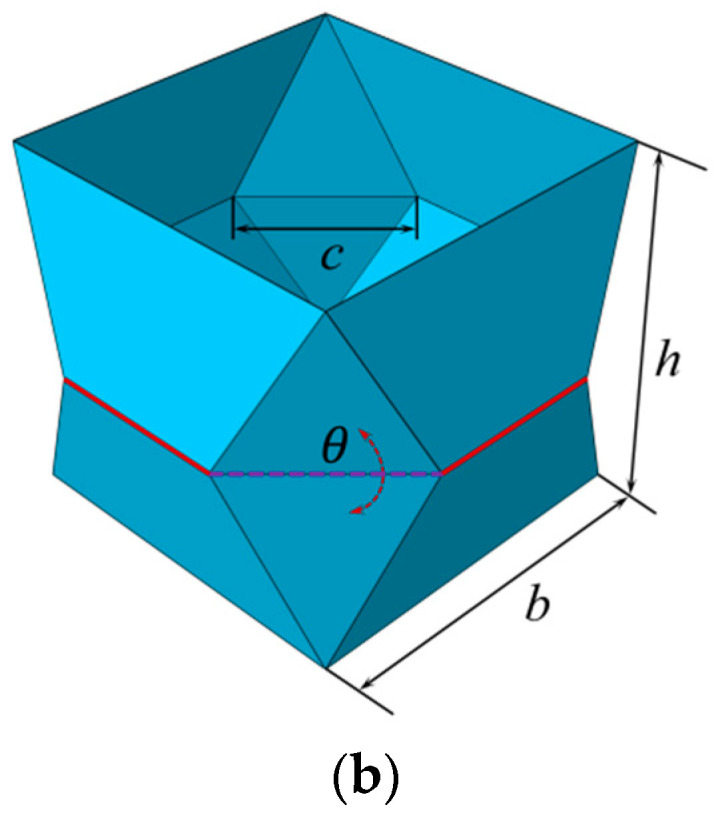
(**a**) The origami pattern of a module for OCB; (**b**) a module of OCB.

The OCB consists of a series of identical units, the detail is displayed in Figure 2. The OCB is made by four pre-manufactured origami patterns known as the diamond folded lobes which are the key geometric feature of origami tubes. The solid lines stand for mountain creases and the dashed lines denote valley creases. A standard OCB module is parameterized by the width of folded lobe *c*, the width of tube *b*, and the unit length *l* [27] (unfolded width of OCB) as shown in Figure 2a. The relationship between different parameters is illustrated by Equation (1):

(1) $$\cos\frac{\theta }{2}\;=\;(\sqrt{2}-1)\frac{c}{l}$$

where θ is the dihedral angle of folded lobe in Figure 2b. The upper and lower ends of a unit are two same squares with the length of the sides equal to *b*. Thus, a longer OCB can be assembled by stacking several modules end to end. Under these circumstances, the height of an OCB is *H* = *Mh*, and the length of an OCB *L* equals to *Ml*, where *M* is the number of units in one OCB and the height of a unit is *h* [35]. Hence, the relationship between *H* and *L* can be calculated as follows:

(2) $$\sin\frac{\theta }{2}=\frac{h}{l}$$

The geometry constraint *c* ≤ *b* is executed in this study, to ensure that the structure pertains to the tubular structure. In addition, other geometry constraints are as follows: $c\leq \left(\sqrt{2}+1\right)l$ since cos θ/2≤1.

### 2.2. Materials

The polylactic acid (PLA/1.75 mm) filament is used in this research, which is manufactured by Shenzhen Creality 3D Technology Co, Ltd. (Shenzhen, China) with a density of 1.25 g/cm^3^. The sample is formed by a fused filament fabrication (FFF) 3D printer CR-10S (Shenzhen Creality 3D Technology Co, Ltd., Shenzhen, China) (Figure S1a, Supplementary Materials). FFF is one of the widely used technologies of 3D printing, in which filaments are melted and squeezed through a heated nozzle and are deposited layer by layer onto the hotbed where layers are fused and form a three-dimensional model. The sample geometry is designed in accordance with the ASTM-D412 standards (Figure S2, Supplementary Materials). In the 3D printing process, the nozzle temperature and hot-bed temperature is set to 210 °C and 60 °C, respectively. The printing speed of the samples is 50 mm/s, packing density is 100%, and layer height is 0.2 mm. In addition, to ensure that each layer of the specimen has the same properties, a building platform is first printed on the hot bed as a support system, and then the sample is printed on the building platform (Figure S3, Supplementary Materials). Meanwhile, to ensure the same printing environment, each sample is printed in an enclosed space and the printer will cool down for a period of time after printing a sample. Due to the FFF printed method, the mechanical properties of the 3D print structure will be affected by certain factors such as the layering direction and nozzle path. In this study, the samples in the 0° and 90° orientations are printed. The angle at which the printing direction is perpendicular to the length direction is defined as 90°.

A tube is manufactured using a fused deposition model 3D printer: OCB, the size of tube is performed in Figure S4 (Supplementary Materials), in which the height, width, and thickness are respectively *H*, *b*, and *t*. For OCB, the width of folded lobe *c* = 30 mm. In this research, the printing process parameters of the OCB are the same as the tensile sample.

### 2.3. Mechanical Test

#### 2.3.1. Tensile Test

To obtain the constitutive model of PLA material, a quasi-static uniaxial tensile test is carried out on the sample by a precision electronic universal material testing machine AGS-X (Shimadzu Instruments Co, Ltd., Suzhou, Jiangsu, China) (Figure S1b, Supplementary Materials). The sample stretching is completed in a temperature environment chamber with a heater and a circulating fan to characterize the mechanical property of printed material at three different temperatures: 30 °C, 40 °C, and 50 °C. Before the sample is stretched, each sample is kept at the test temperature for 3 min to ensure that the required test temperature is reached. The samples at each temperature are tested in more than 5 groups to ensure the reliability of the experiment. In addition, tensile experiments use an electronic extensometer to measure the deformation of the samples and load at a rate of 5 mm/min.

#### 2.3.2. Quasi-Static Uniaxial Compression Test

For OCB, the AGS-X universal testing machine with a temperature chamber is used to perform quasi-static uniaxial compression tests, the compression displacement is 65 mm. To evaluate the influence of temperature on the energy absorption boxes, the entire experimental procedure is carried out at three temperatures: 30 °C, 40 °C, and 50 °C. The displacement and compressive force data are recorded by the machine data acquisition system, and video is used to document the compression process. To calculate energy absorption and impact resistance, initial peak force (*F_max_*), average force (*F_m_*), and *SEA* are selected as evaluation criteria. The absorbed energy is the area under the force-displacement curve.

#### 2.3.3. SEM

**Figure 3:**
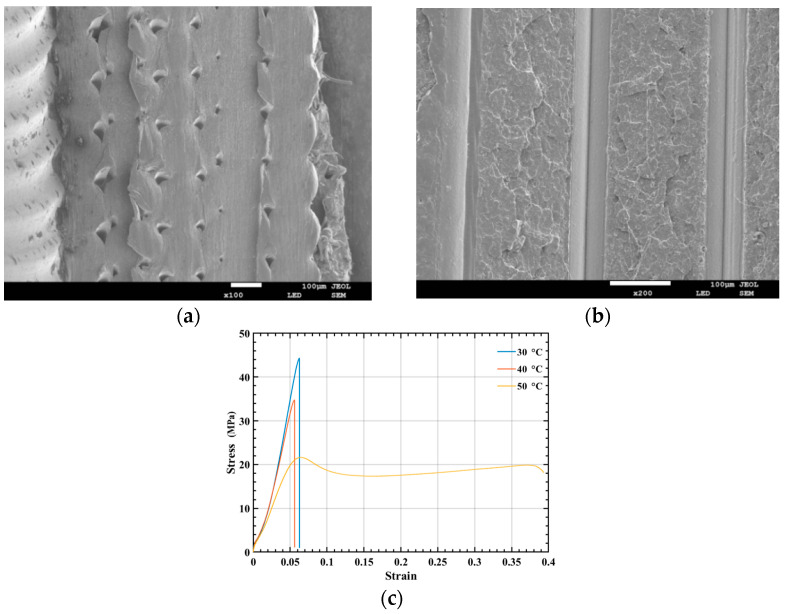
(**a**) The fracture surface of the sample at 0° printing direction; (**b**) the fracture surface of the sample at 90° printing direction; (**c**) the true stress-strain curve of PLA tensile sample at different temperatures.

In order to better study the fracture mechanism of the energy-absorbing box, the fracture surface is analyzed by scanning electron microscope JSM-7800F (JEOL, Tokyo, Japan). The SEM accelerating voltage is 15 KV, and the micrographs are taken at ×100 and ×200 magnification. Figure 3 is the fracture surface of the sample at 40 °C temperature in the 0° and 90° printing directions. According to the two figures, it can be found that although there are some air gaps between the molten material lines, the material can be fused well. The air gap is an intrinsic feature of FFF 3D printed samples [36], and its existence is not affected by the grating orientation. From the topography of the fractured surface, which exhibits an uneven and rough pattern, it can be inferred that the failure of the sample in Figure 3a is mainly due to the fracture of the fuse rather than the fracture of the interlayer adhesion, while in Figure 3b, it is due to the fracture of the interlayer bond, which manifests as a brittle fracture. In addition, the fracture mechanism of the energy-absorbing box at 40 ℃ is a brittle fracture. Therefore, this paper selects the constitutive model with the printing direction of 90° and its true stress-strain curves at different temperatures are presented in Figure 3c.

### 2.4. Numerical Simulation Method

#### 2.4.1. The Damage Models

From the PLA tensile test and tensile stress-strain curve, it can be seen that there is damage in the tensile process of the material. To accurately describe the tensile process of the material, this article is based on the Mazars damage constitutive model, a damage model with damage parameter *D* established. Using the concept of effective stress, under uniaxial tensile load [37,38]:

(3) σ=(1−D)Eε 

σ and ε stand for tensile stress and strain, respectively. E represents the Young’s modulus. *D* is the damage parameter evolving between 0 and 1.

Damage *D* is expressed by the following equation [38]:

(4) $$D=\left\{\begin{array}{cc} 0, & (0\;\leq \;\varepsilon \;\leq \varepsilon_{f})\\ 1-\frac{\varepsilon_{f}\;(1-C_{1})}{\varepsilon }-\frac{A_{1}}{\exp\;\left[B_{1\;}(\varepsilon -\varepsilon_{f})\right]}, & \left(\varepsilon \;>\;\varepsilon_{f}\right) \end{array}\right.$$

$\varepsilon_{f}$ indicates the initial damage strain, $A_{1}$, $B_{1}$, $C_{1}$ is the material constant. The maximum plastic equivalent strain criterion is adopted; when the equivalent plastic strain of the material element in the simulation exceeds the given failure threshold, the element is deleted.

#### 2.4.2. The Realization Process of VUMAT Subroutine

The total strain ε can be divided into two parts. $\varepsilon^{e}$ is the elastic strain that obeys Hooke’s law, and $\varepsilon^{p}$ is the plastic strain that conforms to the law of plasticity. It can be written as follows [39]:

(5) $${\varepsilon =\varepsilon }^{e}{+\varepsilon }^{p}$$

After time iteration, the calculation formula of elastic strain can be written as [40]:

(6) $$\varepsilon^{e}{=\varepsilon }_{t}^{e}+\Delta \varepsilon^{e}{=\varepsilon }_{t}^{e}+\Delta \varepsilon -\Delta \varepsilon^{p}$$

Δε, $\Delta \varepsilon^{p}$, and $\Delta \varepsilon^{e}$ are strain increment, plastic strain increment, and elastic strain increment, respectively.

Elastic Hooke’s law is expressed as [41]:

(7) $$\sigma =2G\left(\varepsilon_{t}^{e}+\Delta \varepsilon -\Delta \varepsilon^{p}\right)+\lambda \mathrm{Tr}\left(\varepsilon_{t}^{e}+\Delta \varepsilon -\Delta \varepsilon^{p}\right)I$$

where G represents the shear modulus.

When the material is in the linear elastic stage, use the linear elastic formula to solve the trial stress [42]:

(8) $$\sigma^{\mathrm{tr}}=2G\left(\varepsilon_{t}^{e}+\Delta \varepsilon \right)+\lambda \mathrm{Tr}\left(\varepsilon_{t}^{e}+\Delta \varepsilon \right)I$$

$\sigma^{\mathrm{tr}}$ and $\sigma^{{\mathrm{tr}}^{\prime }}$ represent the trial stress and the trial deviatoric stress, respectively, and use $\sigma_{1}$, $\sigma_{2}$, $\sigma_{3}$ to represent the three stress components in the principal direction. $\sigma^{{\mathrm{tr}}^{\prime }}$ is defined as [43]:

(9) $$\sigma^{{\mathrm{tr}}^{\prime }}{=\sigma }^{\mathrm{tr}}-\frac{1}{3}\left(\sigma_{1}{+\sigma }_{2}{+\sigma }_{3}\right)$$

Von Mises equivalent stress based on pure elastic behavior is defined as follows [40,41]:

(10) $$\sigma_{e}=\sqrt{\frac{3}{2}\sigma^{\prime }:\sigma^{\prime }}\;\sigma \;{=\sigma }^{\mathrm{tr}}-2G\Delta \varepsilon^{p}$$

Generally, the plastic flow f is [44]:

(11) $${f=\sigma }_{e}-\sigma_{y}$$

The plastic flow f accounting for the Mazars damage model can be expressed as [45,46]:

(12) $${f=\sigma }_{e}^{\mathrm{tr}}-3G\Delta p-\sigma_{y}\;$$

where Δp is the equivalent plastic strain increment.

The above nonlinear equation can also be expressed as

(13) $$f+\frac{\partial f}{\partial \Delta p}\;d\Delta p+\cdots =0$$

(14) $$f+\frac{\partial f}{\partial \Delta p}\;d\Delta p+\frac{\partial f}{\partial p}\;\mathrm{dp}=0$$

Solve the following equations by Newton’s method:

(15) $$d\Delta p=\frac{\sigma_{e}^{\mathrm{tr}}-3G\Delta p-\sigma_{y}}{3G+h}$$

where the plastic modulus h can be defined as [47]

(16) $$h=\frac{\partial \sigma_{y}}{\;\partial \Delta p}+\frac{\partial \sigma_{y}}{\partial p}=\frac{\partial \sigma_{y}}{\partial p}$$

Calculate Δp:

(17) $$\Delta p^{\left(k+1\right)}=\Delta p^{\left(k\right)}+d\Delta p$$

Express $\Delta \varepsilon^{p}$ and $\Delta \varepsilon^{e}$ through Δp, the equation is as follows [42]:

(18) $$\Delta \varepsilon^{p}=\frac{\;3}{2}\Delta p\frac{\sigma^{{\mathrm{tr}}^{\prime }}}{\sigma_{e}^{\mathrm{tr}}}$$

(19) $$\Delta \varepsilon^{e}=\Delta \varepsilon -\Delta \varepsilon^{p}$$

Solve for the stress increment, which can be expressed by the elastic strain increment as [41]

(20) $$\Delta \sigma =2G\Delta \varepsilon^{e}+\lambda \mathrm{Tr}\left(\Delta \varepsilon^{e}\right)I$$

The specific program flow is shown in Figure S5 (Supplementary Materials).

#### 2.4.3. Verification of VUMAT Subroutine

To verify the effectiveness of the VUMAT program, the proposed constitutive model using PLA material is implemented through the user material subroutine and imported into the finite element software ABAQUS/Explicit (6.14, Providence, RI, USA). In this study, the constitutive model printed in 90° directions is selected, and the specific material parameters are shown in Table 1. The subroutine is used to simulate the tensile process of the sample, and its dimension is the same as that of the 3D-printed test sample. During simulation, the mesh uses hexahedral elements (C3D8R) and the mesh size is 1 mm (Figure S6, Supplementary Materials). The displacement boundary condition is used as the loading process, the forced displacement is applied in the axial direction of the mobile zone, and the other side of the sample is clamped.

**Figure 4:**
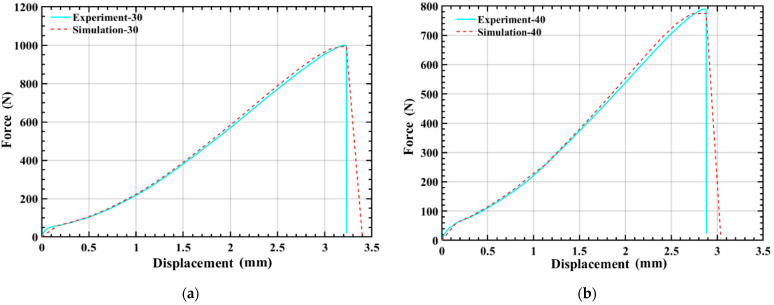
Force-displacement relationships of 3D printed PLA under uniaxial tension at temperature of (**a**) 30 °C and (**b**) 40 °C.

The tensile force-displacement curve predicted by the finite element model is compared with the experimental data, as shown in Figure 4. Obviously, the force-displacement curve of the numerical simulation is in excellent agreement with the obtained result through the experiment, and the load peak value and failure stroke of the sample during the test can be predicted. Hence, the constitutive model proposed by the user material subroutine can reproduce the experimental behavior of the PLA tensile sample in test with good accuracy.

#### 2.4.4. Finite Element Modeling (FEM)

**Figure 5:**
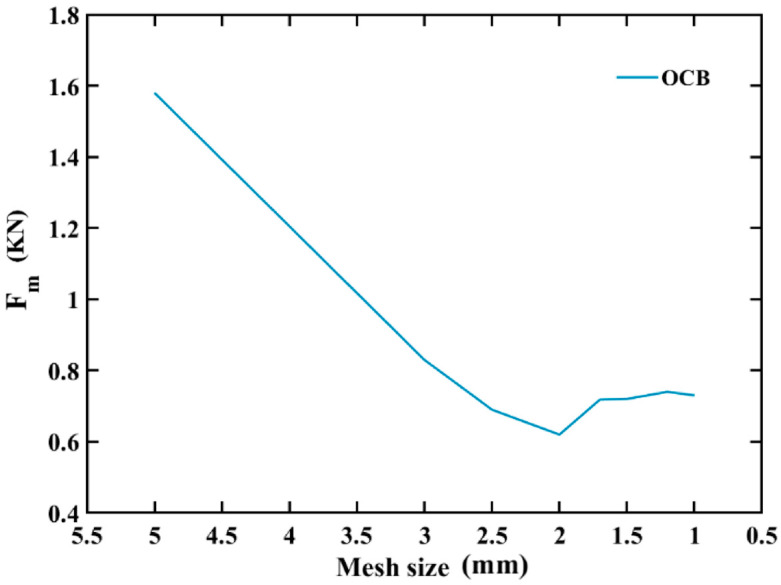
Influence of mesh size on average peak force for finite element simulation.

Quasi-static axial compressed tests of the OCB, which are generated by SolidWorks software, are simulated by using the finite element software ABAQUS/Explicit. During simulation, the top and lower planes are two parallel rigid plates and the models are put between two parallel rigid plates [48]. The upper rigid plate moves in the axial direction to compress the models, and the bottom rigid plate is totally fixed to support the models. Smooth amplitude, which is defined to build in ABAQUS, is applied to control loading rate. The final compression distance is 65 mm, which is identical to that in the quasi-static axial experiments. Moreover, to avoid initial penetration, there is a clearance of 0.3 mm between the upper rigid plate and the top edge of the model as well as between lower rigid plate and the bottom edge of the model. In addition, surface-to-surface contact is adopted between the two rigid plates and the model, and self-contact is applied to simulate the entire model, the coefficient of friction is defined as 0.25, as demonstrated in Figure S7 (Supplementary Materials). All the numerical models are meshed by shell element (S4R) at the same time to ensure the accuracy of the OCB numerical simulation, the mesh sensitivity is studied. It can be seen from Figure 5 that when the global mesh size is between 1.5 mm and 1 mm, the mean crushing force *F_m_* is in a relatively stable state. Therefore, considering the simulation time and simulation accuracy, a global mesh size of 1.2 mm is selected for analysis, except that the platen and base plates are discretized by using a 4-node rigid element (R3D4) with a mesh size of 6 mm. To ensure the hourglass effect can be ignored, the ratio of artificial energy to internal energy should be controlled below 5%. Furthermore, in order to ignore the effect of strain rate, the analysis time of 0.65 s is used in the numerical simulation, and the ratio of kinetic energy to internal energy is also less than 5%. The material property of OCB in this study is obtained by conducting axial tensile tests of the test sample of PLA, and input into ABAQUS by using user-defined material subroutine VUMAT. The values of main mechanical properties, such as density, Young’s modulus, Poisson’s ratio, and yield strain, are provided in Table 1. In addition, for comparison, a conventional square tube (CST) is used as a benchmark and the loading conditions are consistent with the OCB.

In order to explore the energy absorption capacity, the force-displacement curves, deformation processes as well as crush mode are analyzed in this section. Additionally, initial peak force (*F_max_*), average force (*F_m_*), crash force efficiency (*CFE*), and *SEA* are generally used as evaluation indexes. The equation is as follows [49,50]:

(21) $$F_{m}=\frac{E_{a}}{\delta }=\frac{\int_{0}^{\delta }F\left(s\right)\mathrm{ds}}{\delta }$$

(22) $$SEA=\frac{E_{a}}{m}=\frac{\int_{0}^{\delta }F\left(s\right)\mathrm{ds}}{m}$$

(23) $$\mathrm{CFE}=\frac{F_{max}}{F_{m}}$$

where δ is the final compression distance, *m* is the total mass of tube, *E_a_* is the total energy, *F_max_* is the maximum load in the original phase of compression.

## 3. Result and Discussion

### 3.1. Experimental and Simulation Analysis

**Figure 6:**
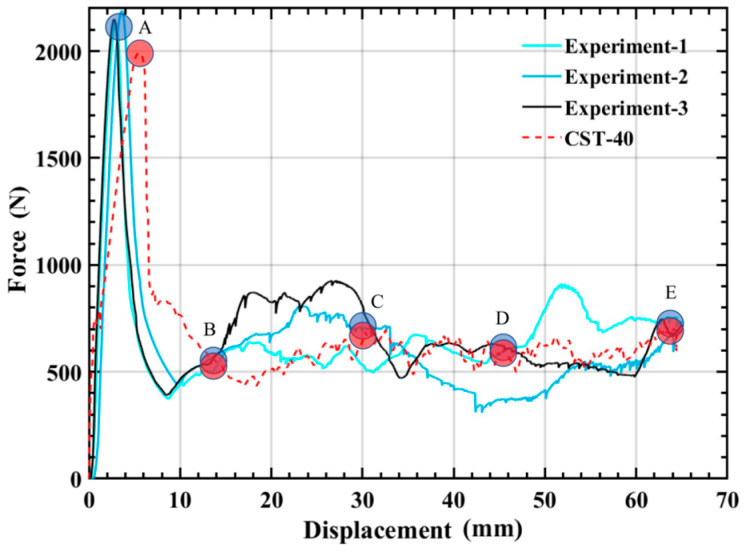
Comparison of experimental and simulated force-displacement curves for CST at 40 °C.

**Figure 7:**
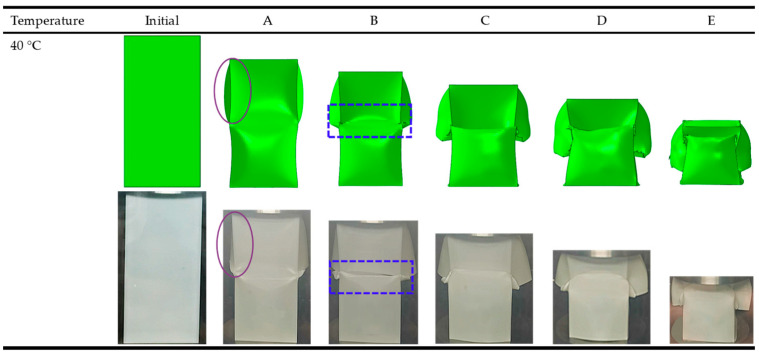
At 40 °C, the experimental and simulation comparison results of the deformation modes of CST.

To validate the numerical simulation method, the simulation and experimental results are compared as displayed in Figure 6. It can be seen that the overall trends are quite similar. When the temperature is 40 °C, for CST, the simulated *F_max_* and *SEA* are 7.59% and 2.36% lower than the experimental ones, respectively. The above detailed data are summarized in Table 2. Figure 6 and Figure 7 compare the experimental and numerical simulation processes of the force-displacement curves and deformation modes about the axial compression for CST at 40 °C and the failure pattern of CST is researched through numerical simulation and compression test pictures. The serial number of each picture in Figure 7 corresponds to the marked point on the curves in Figure 6. It can be seen that the compressive load in Figure 6 began to decrease after reaching the peak value (A), and then the middle of the CST began to fail, which is shown in column B of Figure 7. Due to the failure of the middle part of the tube, the resistance of the compressed tube is low, so the compressive load reaches the lower zone (B, C, D). Meanwhile, the tube continues to fail and the upper part tube buckles, until it compresses to the final area (E).

It is worth noting that certain levels of difference between the simulation and experimental results exist. The main reasons are that the bottom of the experimental specimen is in free contact with the lower surface of the universal testing machine; nonetheless, during the finite element simulation process, the bottom edge of the finite element model and the lower rigid plate are fixed constraints. In addition, the elements that failed in the simulation have been deleted, but there are still crack formation areas in the experiment. This leads to slightly different deformation modes and force displacement curves observed in the simulation and experiment. In summary, simulations can predict and validate experimental results well.

### 3.2. Comparison between Different Types of Tubes

**Figure 8:**
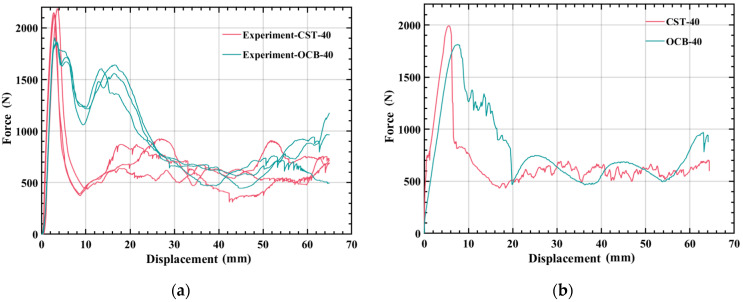
(**a**) Comparison of force-displacement curves of OCB and CST in experiment; (**b**) comparison of force-displacement curves of OCB and CST in numerical simulation.

In this section, the force-displacement curves of experiment and numerical simulation of CST and OCB are drawn, and the results of experiment and numerical simulation are calculated as shown in Figure 8 and Table 3, respectively. At a temperature of 40 °C, the SEA of the OCB simulation and experiment increased by 13.61% and 27.49%, respectively, compared with CST. This can be explained by the deformation process. Obviously, four dynamic inclined hinge lines are formed near each corner of the OCB compared to the CST. It is worth noting that, for CST, only one dynamic inclined hinge line is formed at each corner, as shown in column A of Figure 7, more dynamic inclined hinge lines cause more energy absorption. In addition, *F_max_* of OCB simulations and experiments is reduced by 8.94% and 15.01%, compared with CST, respectively. This is because the direction of the folded blades and the trapezoidal lobes the OCB is inclined, resulting in a significant reduction in axial stiffness compared to CST. Therefore, OCB improve the energy absorption performance of the tube and is better than CST. Next, we will focus on the analysis of OCB.

### 3.3. The Effect of Temperature on OCB

**Figure 9:**
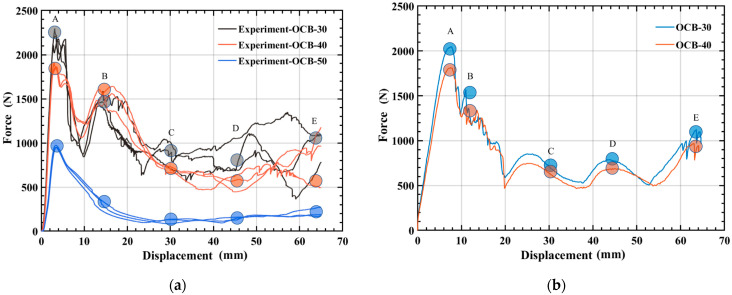
Force-displacement curves of OCB: (**a**) experimental force-displacement curves; (**b**) numerical force-displacement curve.

**Figure 10:**
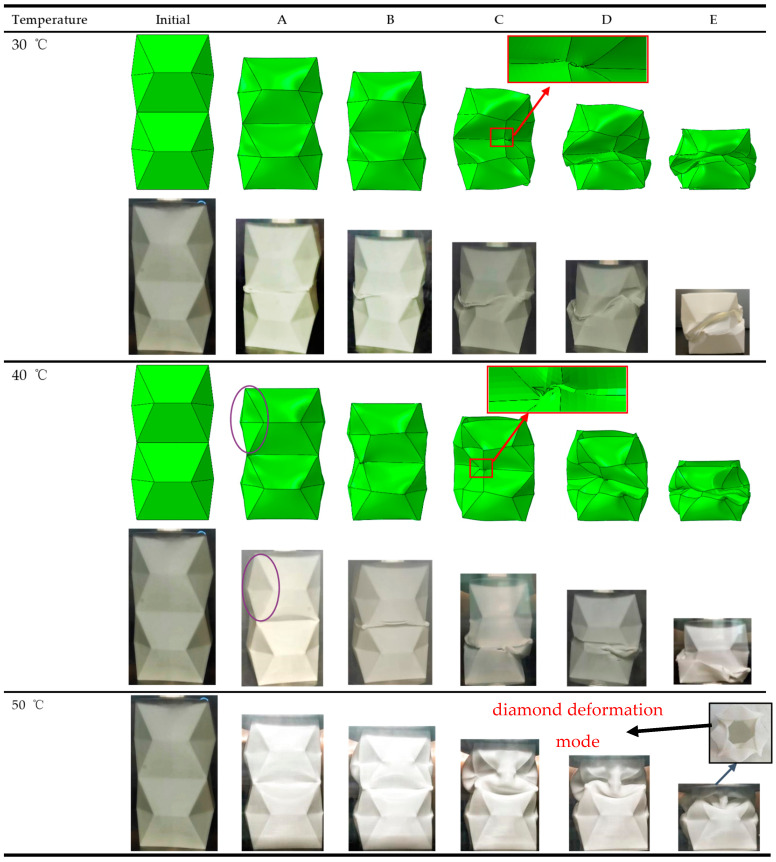
At 40 °C, the experimental and simulation comparison results of the deformation modes of OCB.

Based on force-displacement curves in Figure 9 and the formulas in Section 2, Table 4 shows the experimental data at 30 °C and 40 °C. All the experimental and numerical results can be calculated and are shown in Table 5 and Table 6. It is noted that the energy absorption performance of OCB is closely related to temperature. As the temperature increased from 30 °C to 40 °C, the *F_max_* and *SEA* of OCB decreased by 16.08% and 9.04%, respectively, and the CFE increased by 8.89%. For simulation, *F_max_* and *SEA* decreased by 11.41% and 10.37%, respectively, and CFE increased by 2.33%. Therefore, the energy absorption capacity of OCB at 40 °C is better than that of OCB at 30 °C. The main reason is that the damage of OCB is smaller at 40 °C. In addition, in Figure 10, several key instants are highlighted, the initial peak load (A), the second peak load (B), the lower load area (C, D), and the final area (E). During the axial compression of OCB, when the peak load (A) is reached the stress at the connection of the two modules is the largest, and since the ends of the folded blade are sharp, this area is very sensitive to geometric defects. Therefore, after further axial compression is applied, the left side of the sharp corners at the junction of the two modules begins to fail and cracks are formed, and the compressive load decreases, while the other areas remain undamaged. As the deformation continues, the com-pressive load reaches the second peak (B). After that, the damage further increases and the crack expands continuously, which leads the compression load to drop to the lower area (C, D), and finally the model continues to deform until the final zone (E). The degree of local cracking is different at 30 °C and 40 °C, which can be seen from the point C of the simulation as shown in Figure 10.

**Figure 11:**
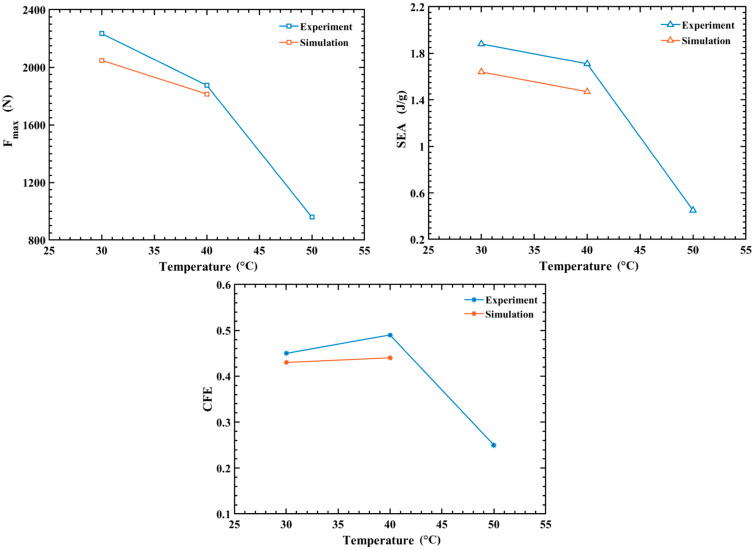
Experimental and simulated energy absorption indexes at different temperatures.

**Figure A1:**
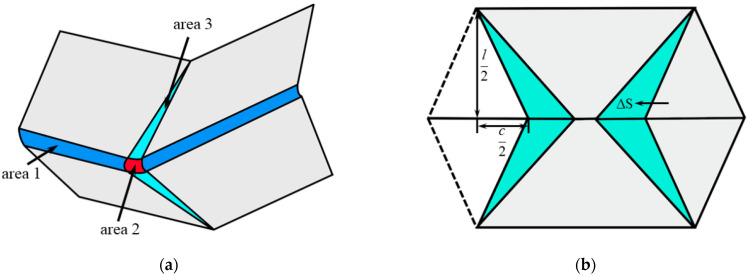
(**a**) The area of energy absorption; (**b**) the moving of the hinge lines.

*F_max_*, *SEA*, and *CFE* drop sharply at 50 °C, as shown in Figure 11 and Table 5. This is mainly because the stiffness of the model made of PLA material declines dramatically at high temperature. PLA material has a complex constitutive relation at high temperature and there is still no reliable method to realize in finite element software, so finite element analysis is not performed at 50 °C, but all OCB in experiments occur in diamond deformation mode (the four sides of the tubes are deformed inward) [35], as shown by E in Figure 10 (the part framed with black wire). Thus, its theoretical solution can be obtained by theoretical calculation. According to the super-folded element theory [51], the energy is mainly absorbed by three parts, namely the bending of the plate about the plastic hinge (area 1), material movement in the annular surface (area 2), and the movement of the conical surface plastic hinge (area 3). The details are shown in Figure A1, in the Appendix A. The detailed formulas of theoretical derivation are shown in (A1)–(A25) in Appendix A [16].

### 3.4. Theoretical Solution of OCB at 50 °C

Table 7 reveals the *F_m_* and *SEA* results of theoretical method and experiment analysis. *F_m_* and *SEA* of theoretical method are 14.74% and 15.09% higher than experiment, respectively. It can be seen that the theoretical results are in good agreement with the experiment analysis results. The error is about 15% which indicates that the theoretical solutions can efficiently predict the experimental results at 50 °C. The main reason for the error is that the influence of the environment on the results is not considered in the theoretical analysis process, so that the theoretical results are larger than the experimental results.

## 4. Conclusions

In this paper, the response process of 3D-printed CST and OCB models of PLA under quasi-static compressive load is studied. In order to accurately predict the mechanical behavior of the model, the PLA stress-strain constitutive relations at 30 °C and 40 °C are obtained experimentally, and the damage criterion-based VUMAT subroutine is embedded in the numerical analysis. Compared simulation with experimental data, the result indicates that the consequence of the simulation coincides with the experiment. Based on data analysis, the energy absorption effect of OCB is superior to CST at a temperature of 40 °C. The theoretical solution is calculated for OCB at 50 °C, and the theoretical results are in good agreement with the experiments. The applicability of theoretical calculations to the 3D-printed PLA model OCB is demonstrated. When comparing the experimental results of the OCB model at 30 °C, 40 °C, and 50 °C, it is clear that the energy absorption effect of OCB decreases with increasing temperature. This reflects the temperature sensitivity of PLA materials.

## Figures and Tables

**Table 1:** Experimental parameters of FFF 3D printed PLA tensile samples.

Temperature	Density	Elastic Modulus (MPa)	Poisson’s Ratio	$A_{1}$	$B_{1}$	$C_{1}$
30 °C	1250 kg/m^3^	2341	0.36	0.19	−8.21	1.38
40 °C	1250 kg/m^3^	1650	0.36	0.26	−7.65	0.68

**Table 2:** At 40 °C, the experimental and simulated comparison results of various energy absorption indicators of CST. (EA is the average of the three experiments).

Temperature	Specimen	$F_{\max}\;\left(N\right)$	$E_{\mathrm{total}}\;\left(J\right)$	$F_{m}\;\left(N\right)$	*SEA* (J/g)	$\Delta F_{\max}$ (%)	$\Delta F_{m}$ (%)	ΔSEA (%)
40 °C	Experiment-CST-1	2131.63	43.48	668.92	1.26	-	-	-
	Experiment-CST-2	2188.70	40.46	622.46	1.17	-	-	-
	Experiment-CST-3	2148.93	44.80	689.23	1.29	-	-	-
	CST-EA	2156.42	42.91	660.15	1.24	-	-	-
	CST-40	1992.64	43.79	673.69	1.27	7.59	2.01	2.36

**Table 3:** Comparison of CST and OCB experimental and numerical simulation results.

Temperature	Specimen	*b* (mm)	*c* (mm)	*H* (mm)	$F_{\max}$ (N)	$F_{m}$ (N)	*SEA* (J/g)	$\Delta F_{\max}$ (%)	ΔSEA (%)
40 °C	CST-EA	60.00	-	120.00	2156.42	660.15	1.24	-	-
	OCB-EA	60.00	30.00	120.00	1874.91	915.88	1.71	15.01	27.49
	CST-40	60.00	-	120.00	1992.64	673.69	1.27	-	-
	OCB-40	60.00	30.00	120.00	1814.40	789.85	1.47	8.94	13.61

**Table 4:** Three sets of experimental data at 30 °C and 40 °C.

Temperature	Specimen	$F_{\max}\;\left(N\right)$	$E_{\mathrm{total}}\;\left(J\right)$	$F_{m}\;\left(N\right)$	*SEA* (J/g)	*CFE*
30 °C	Experiment-1	2296.98	71.58	1101.20	2.05	0.48
	Experiment-2	2223.29	63.17	971.87	1.81	0.44
	Experiment-3	2182.16	61.82	951.14	1.77	0.44
	EA	2234.14	65.52	1008.07	1.88	0.45
40 °C	Experiment-1	1864.73	60.15	925.41	1.73	0.50
	Experiment-2	1906.43	57.19	879.77	1.64	0.46
	Experiment-3	1853.57	61.30	942.46	1.76	0.51
	EA	1874.91	59.53	915.88	1.71	0.49

**Table 5:** Energy absorption indexes at different temperatures.

Temperature	Specimen	$F_{\max}\;\left(N\right)$	$E_{\mathrm{total}}\;\left(J\right)$	$F_{m}\;\left(N\right)$	*SEA* (J/g)	*CFE*
30 °C	OCB-EA-30	2234.14	65.52	1008.07	1.88	0.45
40 °C	OCB-EA-40	1874.91	59.53	915.88	1.71	0.49
50 °C	OCB-EA-50	958.80	15.79	242.92	0.45	0.25

**Table 6:** Experimental and simulated energy absorption indexes at different temperatures.

Specimen	$F_{\max}\;\left(N\right)$	$E_{\mathrm{total}}\;\left(J\right)$	${\;F}_{m}\;\left(N\right)$	*SEA* (J/g)	*CFE*	$\Delta F_{\max}$ (%)	ΔSEA (%)	ΔCFE (%)
Experiment-EA-30	2234.14	65.52	1008.07	1.88	0.45	-	-	-
Experiment-EA-40	1874.91	59.53	915.88	1.71	0.49	16.08	9.04	−8.89
OCB-30	2047.99	57.12	878.77	1.64	0.43			
OCB-40	1814.40	51.34	789.85	1.47	0.44	11.41	10.37	−2.33

**Table 7:** In the quasi-static uniaxial compression experiment at 50 °C, the energy absorption index of OCB, TS is the theoretical solution.

Temperature	Specimen	$F_{\max}\;\left(N\right)$	$E_{\mathrm{total}}\;\left(J\right)$	${\;F}_{m}\;\left(N\right)$	*SEA* (J/g)	*CFE*	$\Delta F_{m}$ (%)	ΔSEA (%)
50 °C	Experiment-1	974.42	15.59	239.85	0.45	0.25	-	-
	Experiment-2	950.02	14.86	228.62	0.43	0.24	-	-
	Experiment-3	951.97	16.91	260.15	0.49	0.27	-	-
	EA	958.80	15.79	242.92	0.45	0.25	-	-
	OCB-TS-50	-	18.52	284.92	0.53	-	14.74	15.09

**Figure A2:**
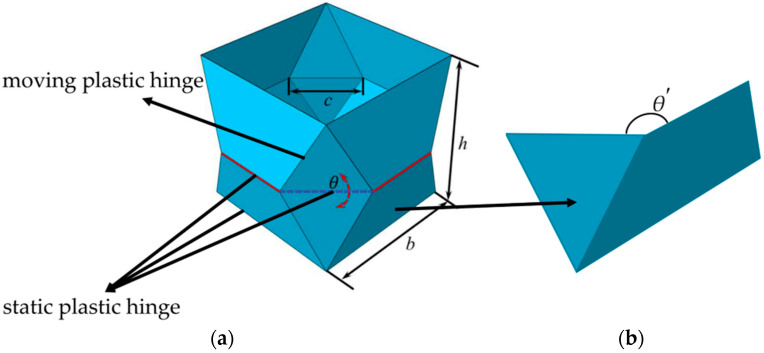
(**a**) A module of OCB; (**b**) $\theta^{\prime }$.

**Figure A3:**
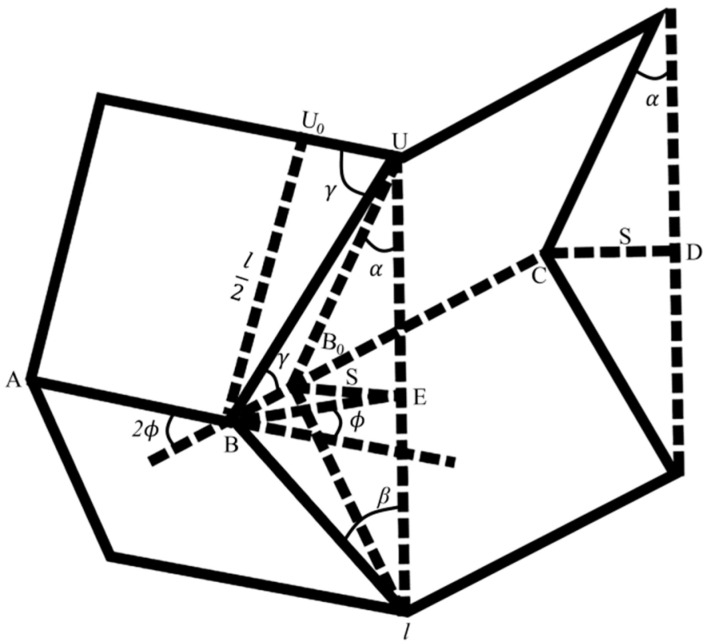
Basic folding element.

**Figure A4:**
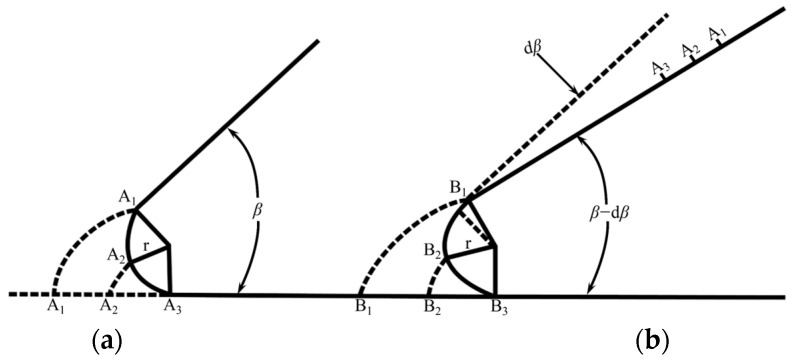
(**a**) Plastic hinge line before moving; (**b**) plastic hinge line after moving.

## Data Availability

The raw/processed data required to reproduce these findings cannot be shared publicly but are available upon request.

## Supplementary Materials

The following supporting information can be downloaded at https://www.mdpi.com/article/10.3390/polym14194135/s1. Figure S1: Equipment of 3D printing and tensile testing for PLA: (a) FFF 3D printer (CR-10S); (b) electronic universal testing machine with temperature chamber (AGS-X); Figure S2: Test sample geometry for tensile testing of 3D-printed parts. Geometry and dimensions specified according to ASTM D412; Figure S3: The direction of printing: α = 90°; Figure S4: Pattern geometry and specific size of OCB; Figure S5: The connection flow chart of ABAQUS and VUMAT for PLA materials; Figure S6: Numerical model of PLA tensile sample; Figure S7: Contact relationship of finite element model:(a) OCB; (b) CST.

## Appendix A. Theoretical Derivation

The energy absorbed by the bending of the plate about the plastic hinge (area 1):

Taking a standard segment of OCB used as the research object, the research object includes the horizontal bending static plastic hinge and the oblique bending moving plastic hinge, as shown in Figure A2a. The inclined moving plastic hinge line stops moving when its included angle with the horizontal line is 45° and becomes a static horizontal hinge [52]. Assuming that the OCB tube has a total of *M* standard segments, the bending angle of the horizontal static plastic hinge of the entire OCB can be expressed:

(A1) $$\theta_{h}=2M\theta -\frac{\theta }{2}\;$$

where θ is the dihedral angle of folded lobe.

The bending angle of the entire OCB inclined to move the plastic hinge line can be simply calculated as:

(A2) $$\theta_{l}{=16M\theta }^{\prime }\;$$

where $\theta^{\prime }$ is the angle between the trapezoidal and triangular faces, as shown in Figure A2b.

(A3) $$\theta^{\prime }=\pi -\cos^{-1}\left[{\cos45}^{o}\times \cos\left(\pi -\theta \right)\right]\;$$

The angle between the moving plastic hinge line and the horizontal line is 45°. When it stops moving, therefore, the length of each oblique moving plastic hinge line can be calculated as:

(A4) $$l_{l}=\frac{c}{{\;2\cos45}^{{}^{\circ}}}\;$$

The bending energy area 1 (*E*_1_) of the OCB can be expressed:

(A5) $$E_{1}{=4b\;\times \;M}_{0}\theta_{h}{+l}_{l}M_{0}\theta_{l}\;$$

The above analysis formulas are substituted into the Equation (A5) [11]:

(A6) $$E_{1}{=4b\;\times \;M}_{0}(2M\theta -\frac{\theta }{2})+\frac{c}{2\cos45{}^{\circ}}{\;\times \;M}_{0}\;\times \;16M\left\{-\arccos\left[\cos\left(45{}^{\circ}\right)\;\times \cos\left(-\theta \right)\right]\right\}\;$$

Energy absorbed by material movement in the annular surface (area 2):

A quarter of a standard OCB is used as the research object, the blue and green area are material movement in the annular surface (area 2) and the movement of the conical surface plastic hinge (area 3), respectively, as shown in Figure A1a, therefore, area 2 ($E_{2}$) and $\mathrm{area}\;3\;\left(E_{3}\right)$ can be calculated as [11,16]:

(A7) $$E_{2}{=8M\;\times \;16I}_{1}\frac{\mathrm{lr}}{2t}M_{0}\;$$

(A8) $$I_{1}=\frac{4}{3\tan\left(\frac{\pi }{8}\right)}\int_{\arcsin(\frac{c}{l}\tan\frac{\pi }{8})}^{\arcsin(\tan\frac{\pi }{8})}\cos\times \{\sin\frac{\pi }{8}\sin\left[\frac{3}{4}\arctan\left(\frac{\tan x}{\sin\frac{\pi }{8}}\right)\right]+\cos\frac{\pi }{8}\;-\cos\frac{\pi }{8}\cos\left[\frac{3}{4}\arctan\left(\frac{\tan x}{\sin\frac{\pi }{8}}\right)\right]\}\mathrm{dx}$$

The upper and lower limits in the integral formula are the initial and final changes of the lateral inclination angle of the super fold element, as shown in Figure A3.

The upper limits in the integral formula are:

(A9) $$\phi_{0}=\frac{\pi }{8}\;$$

(A10) sinα=SUB0=Sl2=c2l2×SBB0=cl×SBB0=cl×tanϕ0=cltanπ8 

The lower limits in the integral formula are:

(A11) $$\gamma =\frac{\;\pi }{4}\;$$

(A12) $$\sin\alpha =\frac{S}{{\mathrm{UB}}_{0}}=\frac{S}{{\mathrm{BB}}_{0}}=\tan\phi_{0}=\tan\frac{\pi }{8}\;$$

Energy absorbed by the movement of the conical surface plastic hinge (area 3):

(A13) $$E_{3\;}=4\;\times \frac{2\Delta S}{r}M_{0}M\;$$

A single moving plastic hinge line is considered (in a flat plate), and its transverse cross-section is shown in the Figure A4, where Figure A4a shows the plastic hinge line before moving, and the Figure A4b shows the plastic hinge line after moving. Therefore, the energy of moving the plastic hinge line can be divided into three parts to calculate. The initial state is on the curved surface, after one bending and the final state is in the *A*_1_*A*_3_ segment on the plane line, the initial state is in the flat state, and the final state is also in the *A*_1_*B*_3_ segment on the flat state after two bending, the initial state is in the plane state, after one bending, the final state is in the *B*_1_*B*_3_ segment on the curved surface [52].

The energy absorbed by *A*_1_*A*_3_ is:

(A14) $${\delta W}_{p}=\frac{\;\bar{A_{1}A_{3}}C}{r}M_{0}=(\pi -\beta ){\mathrm{CM}}_{0}\;$$

where *C* is the width of the plate.

The energy absorbed by *A*_3_*B*_1_ is:

(A15) $${\delta W}^{\prime }{}_{p}=2\frac{\bar{A_{3}B_{1}}C}{r}M_{0}=\;\frac{{2\mathrm{CM}}_{0}}{r}\left(\delta l-\frac{\left(\pi -\beta \right)r}{2}-\frac{\left(\pi -\beta -\Delta \beta \right)r}{2}\right)=\frac{{2C\delta \mathrm{lM}}_{0}}{r}\;-C\left(\pi -\beta \right)M_{0}-C\left(\pi -\beta -\Delta \beta \right)M_{0}$$

where δl is the length of the plastic hinge movement.

The energy absorbed by *B*_1_*B*_3_ can be calculated as follow:

(A16) $${\delta W}^{\prime \prime }{}_{p}=\frac{\bar{B_{1}B_{3}}C}{r}M_{0}=C\left(\pi -\beta -\Delta \beta \right)M_{0}\;$$

Therefore, the total energy absorption is:

(A17) $${\delta W=\delta W}_{p}{+\delta W}^{\prime }{}_{p}{+\delta W}^{\prime \prime }{}_{p}=\frac{2\delta \mathrm{lC}}{r}M_{0}=\frac{\;2\Delta S}{r}M_{0}\;$$

where ΔS is the area of plastic hinge movement as shown in Figure A1b. *r* is the radius that is a curved surface by plastic hinge movement, as shown in the Figure A4. It can be seen from Figure A1b that the expression of ΔS is [52]:

(A18) $$\Delta S=\frac{1}{2}l\left(l-c\right)$$

(A19) $$r=\frac{\sqrt{2}}{4}\;(\Delta {\mathrm{St})}^{\frac{1}{2}}{{(I}_{1}l)}^{-\frac{1}{2}}\;$$

The relationship between *h* and *l* can be calculated as follows:

(A20) $$\sin\frac{\theta }{2}=\frac{h}{l}\;$$

(A21) $$h=\frac{120}{M}\;$$

Substitution of (A8), (A18), (A20), and (A21) into (A13), $E_{3}$ can be expressed as

(A22) $$E_{3}=\frac{{4M}_{0}M}{r}\left\{\frac{14400}{M^{\;2}\sin^{2}\mathrm{arccot}\left[\left(\sqrt{2}-1\right)\frac{\mathrm{cM}}{120}\right]}-\frac{120}{M\mathrm{sinarccot}\left[\left(\sqrt{2}-1\right)\frac{\mathrm{cM}}{120}\right]}\right\}$$

where $M_{0}$ is ultimate bending moment per unit length, $\sigma_{0}$ is the equivalent plastic flow stress of the material, the specific derivation can refer [53,54],

(A23) $$M_{0}=\frac{1}{4}\sigma_{0}t^{2}\;$$

(A24) $$\sigma_{0}=\sqrt{\frac{\sigma_{y}\sigma_{u}}{1+n}}\;$$

where $\sigma_{y}$ is the yield stress of the material, $\sigma_{u}$ is the ultimate stress of the material.

Therefore, the total energy absorbed can be calculated as [11]:

(A25) $$E_{\mathrm{total}}{=E}_{1}{+E}_{2}\left(r\right){+E}_{3}\left(r\right)\;$$
