# Supplementary material for: Experimental and Numerical Investigation of 3D Printing PLA Origami Tubes under Quasi-Static Uniaxial Compression
Source: Polymers (Basel). 2022 Oct 2;14(19):4135. doi: 10.3390/polym14194135 (PMC9573725; doi:10.3390/polym14194135)
Supplement: Supplementary file 1 [file polymers-14-04135-s001.zip › polymers-1950075-supplementary.pdf]

Supporting Information

on

Experimental and numerical investigation of 3D printing PLA origami tubes under quasi-static uniaxial compression

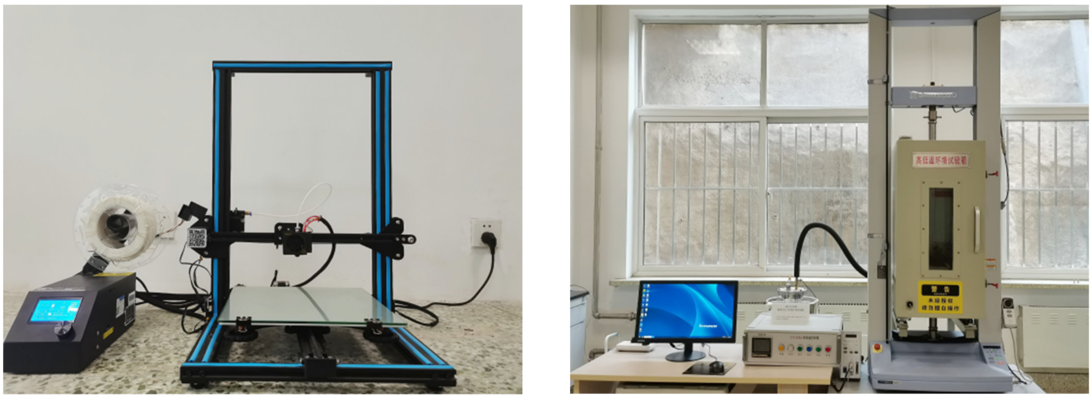

(a) (b)

Figure S1. Equipment of 3D printing and tensile testing for PLA: (**a**) FFF 3D printer (CR-10S); (**b**)electronic universal testing machine with temperature chamber (AGS-X).

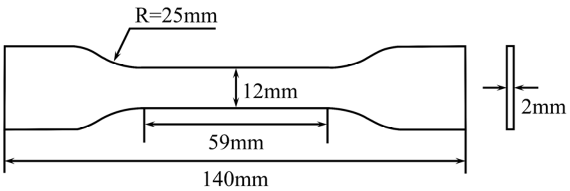

Figure S2. Test sample geometry for tensile testing of 3D printed parts. Geometry and dimensions specified according to ASTM-D412.

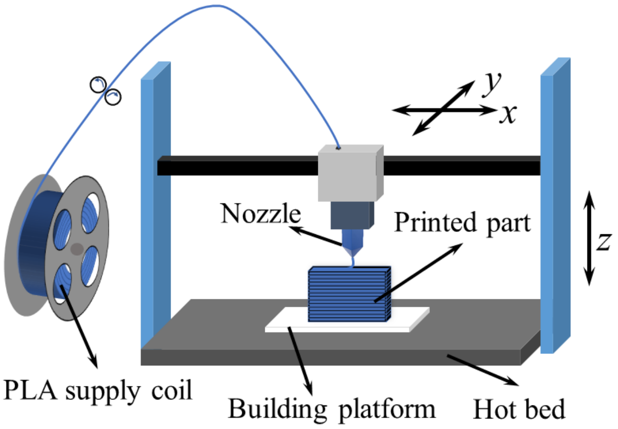

Figure S3. The direction of printing: $\alpha = 90°$.

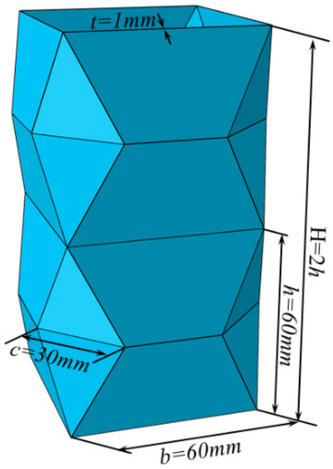

Figure S4. Pattern geometry and specific size of OCB.

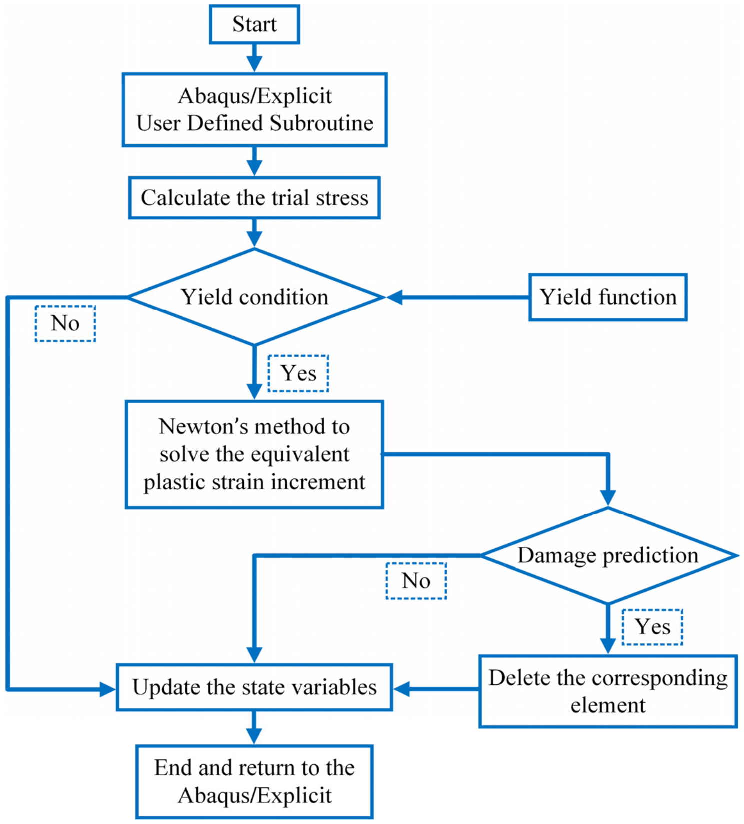

Figure S5. The connection flow chart of ABAQUS and VUMAT for PLA materials.

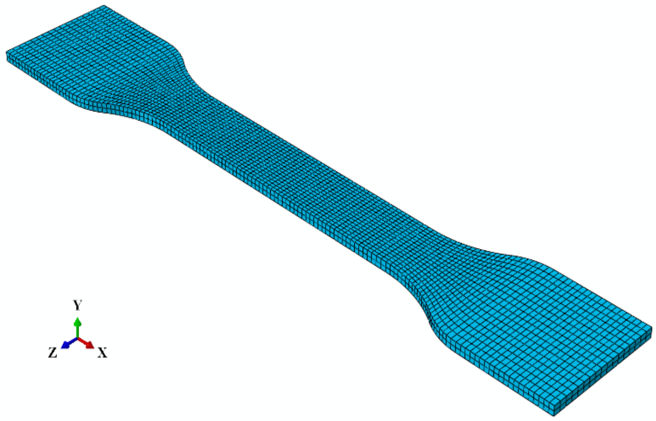

Figure S6. Numerical model of PLA tensile sample.

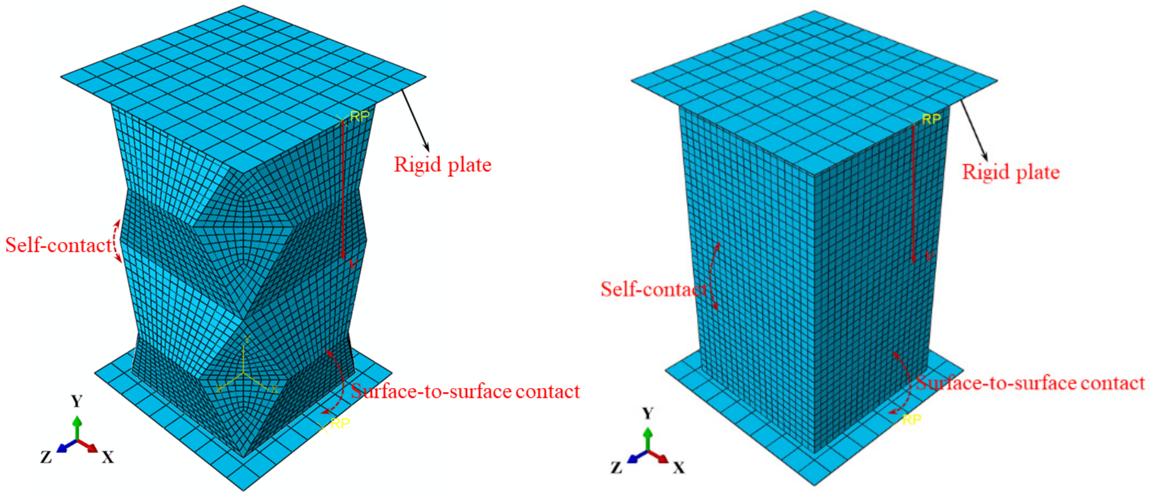

(**a**) (**b**)

Figure S7. Contact relationship of finite element model :(**a**) OCB; (**b**) CST.
